# Cooking Skills and Associated Variables in Public University Students from Northeast Brazil

**DOI:** 10.3390/nu17101606

**Published:** 2025-05-08

**Authors:** Eva Débora de Oliveira Andrade, Manuela Mika Jomori, Rafaela Nayara da Costa Pelonha, José Douglas Bernardino Domingos, Érika Paula Silva Freitas, Ana Paula de Bulhões Vieira, Thaysa B. Cavalcante Brandão, Bruna Merten Padilha, Thaís Souza Passos, Bruna Leal Lima Maciel

**Affiliations:** 1 Post Graduate Program in Health Science, Center for Health Science, Federal University of Rio Grande do Norte, Natal 59078-970, Brazil; evaandradenutrii@gmail.com (E.D.d.O.A.); rafaelanayara52@gmail.com (R.N.d.C.P.); 2 Department of Nutrition, Federal University of Santa Catarina, Florianopolis 88040-900, Brazil; manuela.jomori@ufsc.br; 3 Post Graduate Program in Nutrition, Department of Nutrition, Federal University of Rio Grande do Norte, Natal 59078-970, Brazil; douglasdomingosnutri@gmail.com (J.D.B.D.); erika.freitas@ufrn.br (É.P.S.F.); thais.passos@ufrn.br (T.S.P.); 4 Faculty of Nutrition, Federal University of Alagoas, Maceió 57072-900, Brazil; apaulabulhoes@gmail.com (A.P.d.B.V.); thaysa.brandao@fanut.ufal.br (T.B.C.B.); bruna.padilha@fanut.ufal.br (B.M.P.)

**Keywords:** culinary skills, undergraduates, nutrition

## Abstract

**Background/Objectives:** Cooking skills refer to the confidence, attitude, and application of knowledge to perform culinary preparations. This study aimed to characterize the cooking skills and associated variables in undergraduates from public universities in northeast Brazil. **Methods:** This was a cross-sectional study, with data collected between October 2020 and March 2021. Undergraduates (n = 1203) from two federal institutions participated, the Federal University of Alagoas—UFAL, and the Federal University of Rio Grande do Norte—UFRN, in northeast Brazil. The Brazilian Questionnaire for the Assessment of Cooking Skills and Healthy Eating was used to assess cooking skills. The questionnaire was sent to institutional e-mails and answered online. **Results:** Most students (63.6%) presented high cooking skills, 35.6% intermediate cooking skills, and 0.8% low cooking skills. Logistic regressions showed that students who declared not having learned to cook alone/internet/books/TV programs (AOR = 1.60; 95% CI = 1.175–2.17) were more likely to have low/intermediate cooking skills. The high availability and accessibility of fruits and vegetables (AOR = 0.29; 95% CI = 0.18–0.49) and the high knowledge of cooking terms and techniques were inversely associated with low/intermediate cooking skills (AOR = 0.42; 95% CI = 0.32–0.56). Gender, age, and time available for cooking were not associated with cooking skills. **Conclusions:** Most of the students analyzed presented high cooking skills, and factors such as the way they learned how to cook, the availability and accessibility of fruits and vegetables, and cooking knowledge were associated with their cooking skills. Given this, public policy measures aimed at the university students studied should provide instruction on food preparation, foster culinary knowledge, and encourage and favor the availability and accessibility of fruits and vegetables, positively impacting diet quality and health.

## 1. Introduction

Cooking skills refer to the confidence, attitude, and application of knowledge to carry out culinary preparations, involving meal planning and shopping to the final preparation of food, which can include fresh, minimally processed, processed, or ultra-processed foods [1]. Encouraging the development of cooking skills in adults and young people may be related to better food choices and healthier eating habits, such as increased consumption of fruits and vegetables and a reduction in the consumption of highly processed foods [2,3,4].

Cooking is a complex behavior that encompasses multiple stages and competencies [5,6] and is perceived and practiced differently depending on contextual factors such as income or access to food [7,8]. Cooking more frequently can lead to a decrease in the consumption of fast foods and ultra-processed foods. Depending on the cooking method and ingredients, cooking at home results in lower consumption of processed foods is strongly associated with a better diet quality [9,10]. Preparing meals can also provide more control over the ingredients used, which can have a positive influence on dietary intake and diet-related diseases such as obesity, diabetes, and hypertension [11].

Food preparation is used by societies as a cultural identity, and regardless of the geographic, social, and political differences that separate people, the act of preparing food is inextricably linked to its cultural context, affecting food choices and their transformation [12,13,14]. In addition to the cultural aspect, the act of cooking is also influenced by the local availability of food [15], the age group of those who prepare the food [16,17,18], as well as by issues related to gender. In this sense, while for women, cooking is often associated with a social obligation, for men, it tends to be perceived as an activity focused on leisure or relaxation [19,20,21]. Furthermore, the time available for preparing meals seems to have a direct influence on the development of culinary skills, with the lack of time being related to the increased consumption of ultra-processed and ready-to-eat foods [22,23,24].

In university students, entrance into the academic environment is associated with a reduction in the consumption of fruits and vegetables and an increase in the consumption of ultra-processed foods [25,26]. Eating out, the lack of available time, or the use of quickly prepared ultra-processed foods are barriers that hinder the interest in cooking. Students report having little time to devote to food preparation due to the need to dedicate themselves to academic activities. Thus, university students often do not develop cooking skills and prepare their own food because they believe that the time spent preparing food could be used for other activities [25,27,28].

Additionally, college students are one of the groups most exposed to food insecurity [29,30,31,32]. This insecurity was identified in Brazil, in a cross-sectional study carried out between August 2020 and February 2021, with 4775 undergraduate students from all Brazilian regions, where food insecurity was present in 38.6% of students’ homes [33]. Therefore, promoting culinary skills has proven effective in reducing food insecurity and can enhance the quality of life among university students [34].

Knowing how to prepare food has been associated with greater consumption of fruits and vegetables and reduced consumption of ready-to-eat and ultra-processed foods, thus improving diet quality [35,36,37]. Therefore, understanding which factors are associated with cooking skills is important to develop strategies that favor cooking and take advantage of its full potential as a health behavior that can help improve diet quality and reduce food-related diseases [11]. However, studies aiming to characterize the cooking skills of university students and to identify variables associated with their development are still scarce. Such information is essential to support the formulation of assistance policies targeted at this population. Thus, this study aimed to characterize the cooking skills and associated variables in undergraduate students from Northeast Brazil. The hypothesis under study is that the cooking skills of university students are associated with variables related to food availability, age, gender, time available for cooking, and how these students learned to cook.

## 2. Materials and Methods

### 2.1. Ethical Considerations

The project was approved by the Ethics Committee of the Onofre Lopes University Hospital affiliated with the Federal University of Rio Grande do Norte—UFRN (CAAE 36572420.1.0000.5292, opinion 4.523.788, 02/09/2020), and by the Ethics Committee on Research with Human Beings of the Federal University of Alagoas—UFAL (CAAE: 09427219.5.3002.5013, opinion 4.171.141, 23/07/2020). The study was conducted in accordance with the ethical principles established by the Brazilian National Health Council, which encompass the guidelines set forth in the Declaration of Helsinki.

### 2.2. Study Design

This is a cross-sectional study, with data collected between October 2020 and March 2021, part of the Brazilian multicenter research “Nutrition is in the Kitchen!” [37,38], which studied students from public universities in the south and northeast regions of the country. The present study presents data from the two northeast centers, UFRN and UFAL.

### 2.3. Study Population and Data Collection

This study was conducted with undergraduates from two federal universities in Northeast Brazil. The inclusion criteria were students aged 18 or over who were regularly enrolled in undergraduate courses. In 2019, the two universities had a total of 55,291 undergraduate students. Participants were voluntarily included, without randomization. Invitations to participate and informed consent forms were sent to students’ institutional emails. The project was also promoted through social media to encourage participation and reinforce the purposes and relevance of the research. Those who showed interest in participating by email or social media were directed to the research page on the Google Forms platform. On this page, participants were informed and clarified about the procedures of the study and ethical aspects through the informed consent form, which was available for online acceptance [39,40,41].

**Figure 1:**
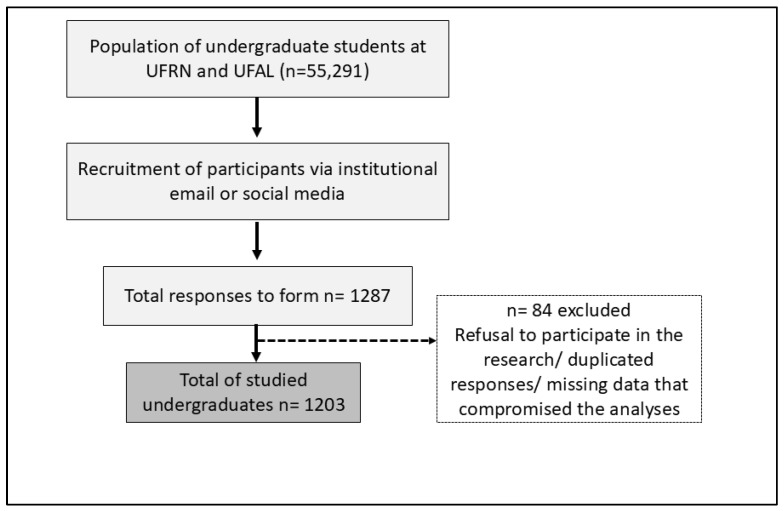
Flowchart of the studied population.

The questionnaire was answered online through the Google Forms platform. A total of 1287 responses were obtained, of which 84 were excluded due to incorrect completion of essential information for the analysis, duplicated responses, and responses from postgraduate students, resulting in a final sample of 1203 participants (Figure 1). The power was calculated a posteriori using the GPower statistical software 3.1, and considering an effect size of 0.1, and an α of 5% the calculated power for the sample of 1203 was 80%.

### 2.4. Sociodemographic Characterization and Meal Preparation

The questionnaire presented a section with 15 questions about sociodemographic and meal preparation/consumption characteristics. The questions also sought information about gender, date of birth, undergraduate course, and the participant’s year of enrollment. They also covered parents’ education, ethnicity, presence of children, and place of origin. Regarding meal preparation characteristics, the available time for cooking per day, who was responsible for meal preparation at home, and the kitchen equipment and utensils available for cooking were registered.

### 2.5. Nutritional Assessment

Weight (Kg) and height (m) data were self-reported by participants in the questionnaire, and the values were used to calculate the body mass index (BMI). After the calculation, BMI was classified according to the World Health Organization [42].

### 2.6. Cooking Skills Assessment

The Brazilian Cooking Skills and Healthy Eating evaluation questionnaire (BCSQ) was used (Supplementary Materials https://www.mdpi.com/article/10.3390/nu17101606/s1). The questionnaire is the result of an adaptation of the “Cooking with a Chef” program and was validated for Brazilian undergraduates [43,44].

The BCSQ presents 36 questions distributed on 7 scales: (1) availability and accessibility of fruits and vegetables—8 items; (2) cooking attitude—4 items; (3) cooking behavior—3 items; (4) self-efficacy in cooking—6 items; (5) self-efficacy in fruits, vegetables and greens consumption—3 items; (6) self-efficacy in using fruits, vegetables, and seasonings—4 items; (7) knowledge of cooking terms and techniques—8 items.

Scales 2–6 were considered for calculating the level of cooking skills, and these were classified as low (20–43 points), intermediate (44–73 points), or high (74–100 points). Availability and accessibility of fruits and vegetables were classified as low (0–2 points), intermediate (3–6 points), or high (7–8 points). The scales 2–6 are punctuated from 1 to 5 points. The knowledge of cooking terms and techniques was classified as high when the participant scored ≥6 points or low when the participant scored <6 points [43,45].

### 2.7. Data Analysis

The data were saved from Google Forms to Microsoft Excel (2013) program for double-blind codification, followed by verification using Excel commands. Regarding the variables related to the characterization of students, the descriptive analysis of categorical variables was carried out by the distribution of absolute (N) and relative (%) frequencies and the discrete and continuous variables by the mean (standard deviation) or median (Q1–Q3), depending on the normality of the data, verified through the Kolmogorov–Smirnov test. The Chi-square test was performed to determine the association of the categorical variables studied with the outcome of low/intermediate cooking skills and high cooking skills. Due to the large sample size, a *p* < 0.01 was considered to avoid type 1 error.

Logistic regression models were calculated to analyze factors associated with cooking skills. First, bivariate analysis was performed, exploring the effect of a single variable on the level of cooking skills (0 = high or 1 = low/intermediate), with non-adjusted odds ratios (OR) and their respective 95% confidence intervals (CI 95%) demonstrated. Then, logistic regression models were calculated, considering cooking skills (0 = high or 1 = low/intermediate) as the dependent variable and independent variables together. The adjustment of the final model chosen was guaranteed by observing the Omnibus test, with *p*-values less than 0.05, and the Hosmer and Lemeshow test, considering *p*-values greater than 0.05. Considering these criteria, the final model included independent variables: gender, age, undergraduate course, time available for cooking per day, availability and accessibility of fruits and vegetables, and knowledge of cooking terms and techniques. The adjusted odds ratios (AOR) and their respective 95% confidence intervals were presented. The analysis was carried out using the statistical program SPSS, version 23 (IBM, New York, NY, USA).

## 3. Results

Most students were female (71.6%), with a median age of 23.0 (20.0–28.0) years, 50.8% declared themselves brown or black, 47.1% white, and 1.5% yellow or indigenous (Table 1). Most undergraduates were enrolled in life sciences (36.7%) and humanities sciences (35.0%) courses, 28.1% of participants were in the first year of undergraduate studies. Most students did not have children (89.9%) and lived mainly with their parents or guardians (66.9%). Regarding nutritional status, 41,1%were overweight or obese (Table 1).

**Figure 2:**
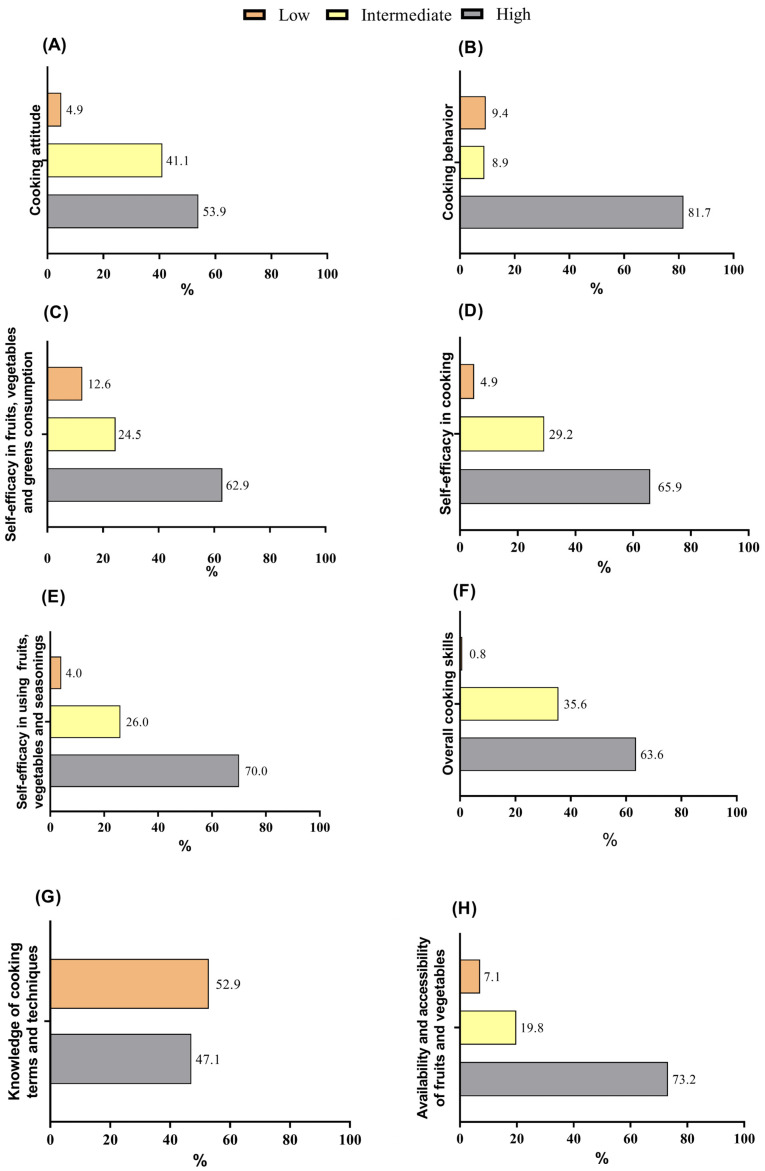
Classification of the studied undergraduates (n = 1203) by cooking skills, according to the Brazilian questionnaire for the assessment of cooking skills and healthy eating (BCSQ). (**A**) Cooking attitude; (**B**) cooking behavior; (**C**) self-efficacy in fruits, vegetables, and greens consumption; (**D**) self-efficacy in cooking; (**E**) self-efficacy for using fruits, vegetables, and seasonings; (**F**) overall cooking skills; (**G**) knowledge of cooking terms and techniques; (**H**) availability and accessibility of fruits and vegetables.

Most participants presented a high cooking attitude (53.9%), cooking behavior (81.7%), self-efficacy in fruits, vegetables, and greens consumption 62.9%), self-efficacy in cooking (65.9%), self-efficacy in using fruits, vegetables, and seasonings (70.0%) and, consequently, high cooking skills (63.6%) (Figure 2). Most students presented low knowledge of cooking terms and techniques (52.9%) (Figure 2G) and high availability and accessibility of fruits and vegetables (69.3%) (Figure 2H).

**Figure 3:**
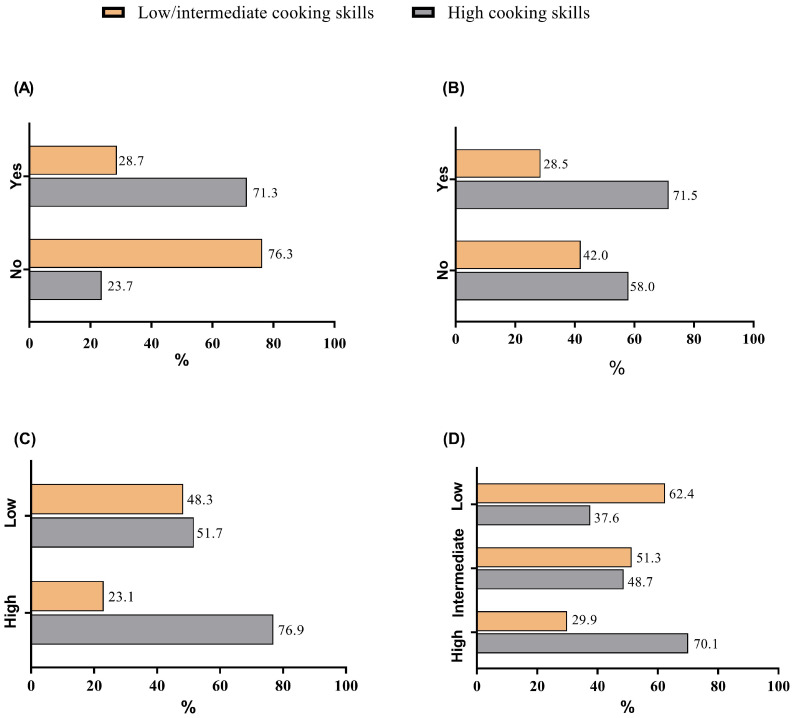
Association of studied variables with low/intermediate and high cooking skills in the studied population (n = 1203). (**A**) Self-declaration of knowing how to cook; (**B**) if the student learned to cook alone/internet/books/TV program; (**C**) knowledge of cooking terms and techniques; (**D**) availability and accessibility of fruits and vegetables.

Among students who reported knowing how to cook, 71.3% showed high cooking skills, while of those who said they did not know, 23.7% showed high skills (Chi-square, *p* < 0.01) (Figure 3A). Most of the students who learned to cook by themselves/internet/books/TV programs showed high cooking skills (71.5%) (Chi-square, *p* < 0.01) (Figure 3B). Among students with high knowledge of cooking terms and techniques, 76.9% showed high cooking skills, while those who had low knowledge 51.7% had high cooking skills (Chi-square, *p* < 0.01) (Figure 3C). Students with high availability and accessibility of fruits and vegetables also showed high cooking skills (70.1%), while only 37.6% of students with low availability showed high cooking skills (Chi-square, *p* < 0.01) (Figure 3D). Although 65.5% of the female and 58.8% of the male students presented high cooking skills, these prevalences were not significant (Chi-square, *p* > 0.01). Most students with obesity (67.8%) and most with normal BMI (63.7%) presented high cooking skills (Chi-square, *p* > 0.01). Age, undergraduate course, year of graduation, if learned how to cook from a family member or in a course, living arrangement, time available to cook/day, and ethnicity were not associated with low/intermediate or high cooking skills.

Among the factors associated with low culinary skills (Table 2), the logistic regression showed that students who did not learn to cook by themselves/internet/books/TV programs were more likely to have low/intermediate cooking skills (AOR = 1.60; 95% CI = 1.18–2.17). Students with high availability and accessibility of fruits and vegetables were 71% less likely to have low/intermediate cooking skills (AOR = 0.29; 95% CI = 0.18–0.49), and students with high knowledge of cooking terms and techniques were 58% less likely to have low/intermediate cooking skills (AOR = 0.42; 95% CI = 0.32–0.56).

## 4. Discussion

This study characterized the culinary skills of undergraduates from universities in Northeast Brazil and identified variables associated with them. Few studies [37,38] aimed to characterize the cooking skills of university students using a validated questionnaire for data collection, as in this study. Although in the present study, most students analyzed presented high cooking skills, 36.4% had low/intermediate cooking skills, allowing the investigation of variables associated with these findings.

Dezanetti et al. [37], also used data from the Nutrition is in the Kitchen! research, evaluated characteristics of meal preparation and consumption among university students from Southern Brazil before and during the COVID-19 pandemic. The authors identified that 70.7% of students had high levels of cooking skills, like the present study, although the population came from a region in Brazil with different cultural and socio-economic characteristics.

In the present study, declaring knowing how to cook, having a high availability and accessibility of fruits and vegetables, and a high knowledge of cooking terms and techniques were associated with high cooking skills. Regarding the self-declaration of knowing how to cook, when students were asked if they knew how to cook, 83.9% of the participants answered yes in the present study. Dezanetti et al. [37] and Bernado et al. [46], also studying university students, identified that 92% and 59% of the participants claimed to know how to cook, respectively. Authors suggest that beliefs about knowing how to cook affect college students, increasing their willingness and availability to prepare their food. Thus, the greater the belief, the greater the ease and recurrence of culinary preparation [22,47].

In our study’s bivariate analysis and logistic regression model, the availability and accessibility of fruits and vegetables were also associated with low cooking skills. Students with high availability of these foods were less likely to have low or intermediate cooking skills. This association can be explained by the nature of these foods, especially vegetables, which require cooking techniques to be incorporated into recipes and preparations. Therefore, having more of these foods available at home improves aspects of self-efficacy in the use of fruits and vegetables, stimulating cooking skills [48,49,50,51].

Previous studies with university students also identified an association between fruit and vegetable consumption and higher cooking skills associated with better food consumption habits [37,52]. Dezanetti et al. [37] identified a relationship between cooking skills and the preparation and consumption of meals among Brazilian university students before and during the COVID-19 pandemic, which is the same context faced by the participants in the present study. The study showed the context imposed by the pandemic encouraged the preparation of meals at home, which may have favored the practice and improvement of cooking skills. These findings reinforce the importance of meal preparation skills as a factor associated with better eating habits and greater consumption of fruits and vegetables among university students. In the present study, the population studied showed high availability and accessibility of fruits and vegetables (73.2%), a result very similar to that found by Dezanetti et al. [37] who, studying a similar population and using the same questionnaire, identified that 73% had high availability and accessibility of fruits and vegetables.

In our study, a high knowledge of cooking terms and techniques was associated with fewer chances for low or intermediate cooking skills. Knowledge of cooking terms and techniques is directly related to improving cooking skills.

This knowledge provides individuals with greater security and confidence in preparing food, in addition to reflecting greater exposure to the topic, whether through practice, reading, or actively searching for information. Studies show that practical educational interventions are effective in this process, promoting advances in both technical mastery and nutritional knowledge. Among the results observed, we highlight the increase in confidence and cooking skills in university students [53], medical students [54], and culinary students [55], reinforcing that the development of learning and understanding of culinary techniques contributes significantly to improving cooking skills.

Regarding the means of learning how to cook, students who reported not having learned how to cook on their own/internet/books/TV programs were 1.60 times more likely to have low or intermediate cooking skills in the present study. This fact can be understood because learning cooking skills through the internet, books, and television programs has proven to be effective, with each resource having specific advantages [56]. Online videos and television programs offer dynamic visual stimuli, which encourage engagement and a practical understanding of culinary procedures. In turn, books provide a more structured approach, allowing for theoretical in-depth study and systematic consultation. The effectiveness of these methods is related to both individual learning preferences and the quality of the materials used. The articulation between these strategies and formal education can enhance the development of cooking skills and contribute to the adoption of healthier eating habits [57,58,59].

Other factors such as income and ethnicity were not associated with the level of cooking skills in the present study. Different from the initial hypothesis of the study, that the cooking skills of university students were associated with variables related to food availability, age, gender, time available to cook, and how these students learned to cook, after the analyses, we observed that only the way in which the student learned to cook was associated with cooking skills in university students, the other variables showed no association.

A limitation of the present study is the non-randomization in the sampling process, but this fact does not invalidate the associations found. Another limitation was the low response rate of participants. Nevertheless, this rate follows those reported in studies involving similar populations and cross-sectional designs [26,38]. Another potential limitation includes the self-reported nature of sociodemographic data collected through an online questionnaire, but the undergraduate population is understanding. Moreover, the Brazilian Cooking Skills and Healthy Eating evaluation questionnaire was previously validated for online application in Brazilian undergraduates [43,44]. BMI was calculated using self-reported data but was not a primary outcome of the study; data collection took place during the social distancing period, and a previous local study [60] demonstrated a consistent correlation between self-reported and researcher-measured values for BMI estimation. Moreover, our prevalences of overweight (26.4%) and obesity (14.7%) were consistent with other Brazilian population-based studies [61,62,63]. Another limitation is that most participants were female. Thus, gender was included as an adjustment variable in the regression model to minimize confounding related to this characteristic.

The data obtained in this study provided important information about students’ cooking skills and factors associated with the level of these skills. This study’s strengths include using a validated instrument to assess the cooking skills of undergraduate students. Another strength is the large sample size, allowing investigations of multiple variables associated with cooking skills, and aiding in understanding variables that may contribute to or negatively interfere with cooking skills.

Thus, the results of this study can contribute to the development and strengthening of public policies, especially given the scarcity of studies on cooking skills among undergraduate students. It is important that these policies are geared towards the reality of students, respecting local cultures and eating habits, and promoting effective assistance. In this context, universities are strategic fields for the implementation of these public policies, contributing to promoting the health and well-being of students.

## 5. Conclusions

Most of the undergraduates analyzed presented high culinary skills. However, a considerable portion presented low or intermediate cooking skills. Not learning how to cook alone, through the internet, books, or a TV program was associated with low/intermediate culinary skills. On the other hand, students with high availability and accessibility of fruits and vegetables and high knowledge of cooking terms and techniques were also less likely to present low/intermediate cooking skills. Given this, public policy measures aimed at the university students studied should provide instruction on food preparation, foster culinary knowledge, and encourage and favor the availability and accessibility of fruits and vegetables, positively impacting diet quality and health.

## Figures and Tables

**Table 1:** Characterization of the undergraduates studied (n = 1203).

Continuous Variable	Median (Q1–Q3)
Age	23.0 (20.0–28.0)
Categorical Variables	n (%)
Gender	
Male	342 (28.4)
Female	861 (71.6)
Ethnicity	
White	567 (47.1)
Yellow and Indigenous	18 (1.5)
Black and Brown	611 (50.8)
Undergraduate course by field of knowledge	
Humanities Sciences	421 (35.0)
Exact Sciences	338 (28.1)
Life Sciences	442 (36.7)
Have children	
Yes	122 (10.1)
No	1081 (89.9)
Graduation year	
1st Year	338 (28.1)
2nd Year	221 (18.4)
3rd Year	198 (16.5)
4th Year	187 (15.5)
5th Year	124 (10.3)
≥6th Year	99 (8.2)
Living arrangement	
Alone	92 (7.6)
With parents or guardians	805 (66.9)
With partner/child	220(18.3)
With colleagues and others	86 (7.1)
Body mass index (BMI)	
Underweight	102 (8.5)
Normal weight	606 (50.4)
Overweight	318 (26.4)
Obesity	177 (14.7)

**Table 2:** Logistic regression models for low cooking skills and associated factors in university students.

Independent Variables	Low/Intermediate Cooking Skills			
	OR (95% CI)	*p*-Value	AOR (95% CI)	*p*-Value
Age	1.00 (0.98–1.01)	0.473	0.99 (0.94–1.01)	0.363
Gender				
Female	−	−	−	−
Male	1.33 (1.03–1.72)	0.029	1.09 (0.79–1.49)	0.581
Undergraduate course by field of knowledge				
Humanities Sciences	−	−	−	−
Exact Sciences	0.95 (0.71–1.27)	0.710	0.82 (0.57–1.17)	0.266
Life Sciences	0.76 (0.57–1.00)	0.051	0.82 (0.59–1.141)	0.241
Learned how to cook in a course				
Yes	−	−	−	−
No	1.74 (1.09–2.80)	0.021	1.21 (0.74–1.99)	0.454
Learned how to cook alone/internet/books/TV program				
Yes	−	−	−	−
No	1.81 (1.37–2.41)	0.000	1.60 (1.18–2.17)	0.003
Availability and accessibility of fruits and vegetables				
Low	−	−	−	−
Intermediate	0.64 (0.38–1.05)	0.079	0.71 (0.41–1.24)	0.230
High	0.26 (0.16–0.41)	0.000	0.29 (0.18–0.49)	0.000
Knowledge of cooking terms and techniques				
Low	−	−	−	−
High	0.32 (0.25–0.41)	0.000	0.42 (0.32–0.56)	0.000

## Data Availability

The raw data supporting the conclusions of this article will be made available by the authors on request.

## Supplementary Materials

The following supporting information can be downloaded at https://www.mdpi.com/article/10.3390/nu17101606/s1, Brazilian Cooking Skills and Healthy Eating Questionnaire—BCSQ.
