# Supplementary material for: Cooking Skills and Associated Variables in Public University Students from Northeast Brazil
Source: Nutrients. 2025 May 8;17(10):1606. doi: 10.3390/nu17101606 (PMC12113868; doi:10.3390/nu17101606)
Supplement: Supplementary file 1 [file nutrients-17-01606-s001.zip › nutrients-3600577-supplementary.pdf]

Brazilian Cooking Skills and Healthy Eating Questionnaire – BCSQ (Short version)

**Availability and Accessibility of Fruits and Vegetables Index (AAFV)**			
**DIRECTIONS: This section is about the presence of fruits, vegetables and greens in your house during the past week. Please mark YES or NO for EACH question.**			
**1.**	Did you have 100% natural fruit juice (homemade, including fruit polpe or ready whole juice) in your home last week?	Yes	No
**2.**	Did you have fresh fruit in your home last week?	Yes	No
**3.**	Did you have raw or cooked vegetables and greens in your home last week?	Yes	No
**4.**	Did you have leaf vegetables as salad in your home last week?	Yes	No
**5.**	In the last week, were fruit, vegetables and greens visible somewhere in the kitchen?	Yes	No
**6.**	In the last week, was 100% natural fruit juice (homemade, including fruit polpe or ready whole juice) or fresh fruits visible on the shelf of the refrigerator to be easly used as a snack?	Yes	No
**7.**	In the last week, were fresh vegetables and greens visible on the refrigerator to be easly used as snack?	Yes	No
**8.**	In the last week, were there ready vegetables and greens in the refrigerator to be used in a meal?	Yes	No

**Cooking Atitude Scale (CA)**						
**DIRECTIONS: For each item below, indicate the extent to which you agree or disagree with the following statements.**						
		Strongly disagree	Disagree	Neither agree nor disagree	Agree	Strongly agree
**9.**	I do not like to cook because it takes too much my time.	◻	◻	◻	◻	◻
**10.**	Cooking is frustrating.		◻	◻	◻	◻
**11.**	I like testing new recipes.	◻	◻	◻	◻	◻
**12.**	I find cooking tiring.	◻	◻	◻	◻	◻

Cooking Behavior Scale (CB)						
**DIRECTIONS: For the 3 items below, think about your actual cooking habits. Select ONE box for EACH question**						
**How often did you do the following?**		Not at all	1 to 2 times this month	Once a week	Several times each week	About everyday
**13.**	Reheat or use leftovers to eat in another meal	◻	◻	◻	◻	◻
**14.**	Reheating leftovers from a home cooked lunch or dinner meal	◻	◻	◻	◻	◻
**15.**	Using leftovers from a home cooked meal to make a new dish	◻	◻	◻	◻	◻

Self-EFficacy[1] in Fruits, Vegetables and Greens Consumption Scale (SEPC)						
**DIRECTIONS: For each item below, indicate the extent to which you feel confident about performing the following activities. Select ONE box for EACH question.**						
		**NOT at all confident**	**NOT very confident**	**Neither confident nor unconfident**	**Confident**	**Extremely confident**
**16.**	Eat fruits, vegetables and greens every day at lunch and dinner	◻	◻	◻	◻	◻
**17.**	Eat fruits or vegetables and greens as a snack, even if everybody else were eating other snacks	◻	◻	◻	◻	◻
**18.**	Eat the recommended 3 servings of fruits, and 3 of vegetables and greens every day	◻	◻	◻	◻	◻

[1] Self-Efficacy reffers to the individuals' confidence in performing tasks.

Self-Efficacy[1] in Cooking Scale (SEC)

DIRECTIONS: For each item below, indicate the extent to which you feel confident about performing the following activities. Select ONE box for EACH question.

		NOT at all confident	NOT very confident	Neither confident nor unconfident	Confident	Extremely confident
**19.**	Cook from basic ingredients (ex: lettuce head, fresh tomatoes, raw meat)	□	□	□	□	□
**20.**	Follow a written recipe (ex: preparing *vinagrete* sauce with tomatoes, onion, bell pepper, vinars, olive oil, salt peppers)	□	□	□	□	□
**21.**	Prepare dinner with items you have in the moment in your home	□	□	□	□	□
**22.**	Use knife with skills in the kitchen.	□	□	□	□	□
**23.**	Use basic cooking techniques (e.g. washing, peeling, chopping)	□	□	□	□	□
**24.**	Cooking in boiling water	□	□	□	□	□

Self-Efficacy in Using Fruits, Vegetables, and Seasonings Scale (SEFVS)						
**DIRECTIONS: For each item below, indicate the extent to which you currently feel confident in preparing or using the following foods. Select ONE box for EACH question.**						
		NOT at all confident	NOT very confident	Neither confident nor unconfident	Confident	Extremely confident
**25.**	Fresh or frozen vegetables and greens (ex: broccoli, pea)	◻	◻	◻	◻	◻
**26.**	Fruits (ex: orange, watermelon)	◻	◻	◻	◻	◻
**27.**	Herbs (ex: parsley, spring onion)	◻	◻	◻	◻	◻
**28.**	Vinegars	◻	◻	◻	◻	◻

Knowledge of Cooking Terms and Techniques Evaluation

DIRECTIONS: **For following questions (29-34), mark the option that you consider is the most adequated. Select ONE answer for EACH question.**

29. **Cooking potatoes briefly in boiling water and, following, put in cold water to preserve them for long time or avoid their browining is an example of:**

- Blanching
- Scalding
- Broiling
- Don't know

30. **If a recipe asks you to sauté an onion, you should cook it:**

- In a steam cooker with boiling water.
- In a pan with a small amount of hot oil.
- In a pan with a small amount of water.
- * Don't know.

31. **A diced potato should be cut into:**

- Long pieces, in a thin matchstick format.
- Very small pieces, regular sizes.
- Regular pieces.
- Don't know.

32. **Water is simmering when:**

- Steam begins to form.
- Tiny bubbles emmerge and acumulate on the boarders of the pan.
- Bubbles rise rapidly and break on the surface.
- Don't know.

33. **Sweet potatoe is roasted when it is:**

- Cooked without liquid in the oven.
- Cooked with liquid in the pan in the oven.
- Cooked with a small amount of liquid in a covered pan.
- Don’t know.

34. **What is the term used for washing, peeling and slicing foods before beginning to cook?**

- Blanching
- Preparation
- Mise en place
- Don’t know

DIRECTIONS: For questions 35-36, use the recipe bellow to mark the answer you consider is the most adequated. Please select only ONE answer.

Banana and honey Smoothie
1 sliced banana
1 cup (200ml) of cold whole milk
½ table spoon of bee honey

In a blender, crush the banana, the milk and the honey on high speed until homogeneous smooth. Serve immediately. Yield: 1 portion.

35. **To precisely measure 1 cup of milk for this recipe:**

- Set a liquid measuring cup on a plain surface, bend the recipiente with the milk down and pour into the desired level
- Hold a dry-ingredients measuring at eye level and pour the milk from another container to the desired level
- Set a dry measuring cup on a plain surface, bend the recipiente with the milk down into the desired level
- Don’t know

36. **Which is best utensil for precisely measuring the honey in this recipe?**

* 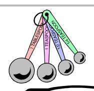
* 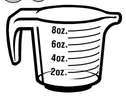

* 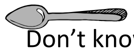
* Don’t know
